# Patients’ and Care Professionals’ Evaluation of the Effect of a Hospital Group on Integrated Care in Chinese Urban Health Systems: A Propensity Score Matching and Difference-in-differences Regression Approach

**DOI:** 10.34172/ijhpm.2023.7897

**Published:** 2023-11-28

**Authors:** Xin Wang, Caiyun Zheng, Yao Wang, Stephen Birch, Yixiang Huang, Pim Valentijn

**Affiliations:** ^1^School of Public Health, Sun Yat-Sen University, Guangzhou, China; ^2^Centre for the Business and Economics of Health, University of Queensland, Brisbane, QLD, Australia; ^3^Department of Health Services Research, Care and Public Health Research Institute (CAPHRI), Faculty of Health, Medicine and Life Sciences, Maastricht University, Maastricht, The Netherlands; ^4^Essenburgh Research & Consultancy, Essenburgh Group, Harderwijk, The Netherlands

**Keywords:** Integrated Care, Hospital Group, PSM–DID Method, C-RMIC-MT

## Abstract

**Background:** A hospital group is an organizational integration strategy that has recently been widely implemented in Chinese urban health systems to promote integrated care. This study aims to evaluate the effect of hospital group on integrated care from the perspectives of both patients and care professionals.

**Methods:** Two cross-sectional surveys were conducted in Shenzhen city of China, in June 2018 and July 2021. All thirty Community Health Stations (CHSs) in the hospital group were included in the intervention group, with 30 CHSs in the same district selected as the control group by simple random sampling. All care professionals within both the intervention and the control groups were invited to participate in the surveys. Twelve CHSs were selected from 30 CHSs in the intervention and the control groups by simple random sampling, and 20 patients with type 2 diabetes mellitus (T2DM) were selected from each of these selected CHSs to participate in the survey by systematic sampling. The Rainbow Model of Integrated Care-Measurement Tool (Chinese version) was used to assess integrated care. Propensity score matching and difference-in-differences regression (PSM-DID) were used to evaluate the effect of the hospital group on integrated care.

**Results:** After matching, 528 patients and 1896 care professionals were included in the DID analysis. Results from care professionals indicated that the hospital group significantly increased technical competence of the health system by 0.771 points, and cultural competence by 1.423 points. Results from patients indicated that the hospital group significantly decreased organizational integration of the health system by 0.649 points.

**Conclusion:** The results suggests that the effect of the hospital group on integrated care over and above routine strategies for integrated care is limited. Therefore, it is necessary to pay attention to implementing professional, clinical and other integration strategies beyond establishing hospital groups, in urban Chinese health systems.

## Background

**Key Messages**

**Implications for policy makers**

- A hospital group has recently been widely implemented in Chinese urban health systems, as an organizational integration strategy for promoting integrated care.
- Our study find that the effect of the hospital group on integrated care over and above routine strategies for integrated care is limited.
- The hospital group significantly improved technical competence and cultural competence from the perspectives of care professionals.
- It is necessary to pay attention to implementing professional, clinical and other integration strategies beyond establishing hospital groups in Chinese urban primary care systems and systems with similar backgrounds in other low- and middle-income countries.

**Implications for the public**

A hospital group is an organizational integration strategy that has recently been widely implemented in Chinese urban health systems. However, can it provide effective integrated care for the public? In this study, evaluation by 532 patients with type 2 diabetes mellitus (T2DM) showed that they were not aware of or feel the changes of integrated care in the hospital group. We hope this study will help to increase public’s awareness of integrated care and engage them to participant in delivery and evaluation of integrated care.

Due to ageing populations and rapid epidemiological change, the number of patients with chronic conditions or multi-morbidity is increasing globally.^1-3^ A continuum of preventive and curative services coordinated across different levels of health systems is required to respond effectively to the needs of these patient groups. However, healthcare systems in many countries currently suffer from fragmentation within and between sectors,^4^ which makes the coordination of health services across different institutions and different care professionals challenging. Integrated care has been suggested as an effective strategy for promoting healthcare coordination, improving quality of care and reducing costs.^5^ In 2016, the World Health Organization (WHO), the World Bank and the Chinese government jointly publishedthe Report *Deepening Health Reform in China* and proposed building a people-centred integrated healthcare system in China.^6^

 China has conducted pilot initiatives to strengthen healthcare in accordance with such an integrated care model. Medical consortia are one of the most widely used strategies for integrated care in pilot initiatives and are formed by different levels and types of health institutions through vertical or horizontal integration of health resources. In 2017, the Chinese General Office of the State Council issued a guideline that encourages all counties and districts to promote the organizational integration of cross-level and cross-type institutions by establishing four types of medical consortia: hospital groups in urban areas, medical associations in rural areas, cross-regional specialist alliances and telecollaboration networks.^7^ By the end of 2019, 1408 hospital groups, 3346 medical associations, 3924 cross-regional specialist alliances and 3542 telecollaboration networks had been established.^8^ Different measures were taken within each medical consortia depending on the characteristics and historical developments of each healthcare system. Examples of integration measures include conducting interdisciplinary and cross-institutional cooperation, integrating health resources, sharing information and reforming health insurance payment methods and salary systems.^9-11^

 As the medical consortia were implemented nationwide, several studies have evaluated its effects on healthcare delivery and patient outcomes. Cai et al explored the effect of a specialist alliance on the health outcomes of cancer patients and showed that the hazard of obtaining favorable outcomes for cancer patients treated in a specialist alliance was significantly lower than that for cancer patients not treated in a specialist alliance.^12^ Several studiesfound that the medical association effectively lowered patients’ blood pressure and hospitalization rates.^13^ Shi et al found that counties that had established a medical association performed better than the control counties in terms of service accessibility, continuity, coordination, comprehensiveness of care and patient satisfaction.^14^ However, some studies did not find significant effects of medical consortia on the quality of primary care provided nor patient satisfaction. Huang et al found that patients enrolled in a Family Physician Integrated Care Program showed no significant decrease in the frequency of emergency department visit or hospitalization rates compared to the control group not enrolled in a Family Physician Integrated Care Program.^15^ Zhang evaluated the effect of a hospital group on patient satisfaction using the Service Quality model and revealed that patient satisfaction with the overall quality of care within a hospital group was low.^16^ Other studies have compared the effects of different medical consortia models. Yuan et al compared the care quality of township health centres (THCs) under three typical integration models and found that the quality of care to patients significantly improved for those in the private hospital-THC integration model, while no changes were observed for patients in either the public hospital-THC integration model or the loose collaboration model.^17^

 Currently, research looking at the effects of medical consortia on healthcare delivery demonstrated mixed findings. Methodologically, healthcare utilization and health outcomes are based on relatively short patient follow-up periods following the establishment of consortia and are thus unable to detect any lags or longer-term policy effects. Moreover, the indicators and measurement tools used in existing research are non-standardized tools and therefore, uncomparable across studies. These tools have mainly been developed according to individual study settings. Meanwhile, there is a knowledge gap in evaluating the effects of medical consortia on integrated care, which is the fundamental aim of establishing medical consortia. Thus, it is necessary to measure integrated care using a standardized and widely used measurement tool, which could support comparisons between different medical consortia or between different health system contexts. This study aims to evaluate the effect of hospital group on integrated care. We improved on previous methodology by using a standardized and widely used measurement tool to assess the perspectives of patients and care professionals on integrated care and to explore the heterogeneity of the evaluation among care professional groups and among patient groups, respectively. We hypothesize that hospital group promote both the implementation of other routine strategies for integrated care and the achievement of integrated care. Patients’ evaluations of integrated care may vary on their socio-demographic and healthcare-related factors, and care professionals’ evaluations of integrated care may vary on their socio-demographic and occupational factors.

## Methods

###  Study Setting

 The government of Shenzhen city has been building a people-centred integrated healthcare system to serve 12.52 million citizens since 2017. The hospital group in Longgang district, Shenzhen, was established in July 2018. Three district-level hospitals and 30 community health stations (CHSs) were vertically integrated into the hospital group to implemented integrated care model. The remaining 60 CHSs in the same district maintain the routine care model. According to the nationwide Equalization of Essential Public Health Service program, all CHSs in Longgang district have established family physician teams. The teams are comprised of general practitioners (GPs), nurses and public health physicians to provide screening, diagnosis, treatment and follow-up management for patients with diabetes and hypertension. However, the routine care model in the 60 CHSs remained treatment-oriented, and lacked coordinated referral mechanism across hospitals.

 Beyond the routine strategies in all CHSs, the Longgang Hospital Group has launched a package of integration strategies at organizational levels. (1) The hospital group integrated leadership and management of administration, finance, healthcare delivery, personnel, drugs, medical equipment, and health information in the 3 hospitals and 30 CHSs. (2) A well-coordinated two-way referral system was established to ensure that patients could conveniently referred from CHSs to hospitals for complex services or referred back to CHSs for rehabilitation and disease management.

###  Study Design and Sampling

 This study uses a quasi-experimental study based on the hospital group. Thirty CHSs in the hospital group formed the intervention group, and 30 CHSs of the remaining 60 CHSs in Longgang district were selected by simple random sampling as the control group. All care professionals in the intervention and control groups were invited to participate in the survey. We selected patients with type 2 diabetes mellitus (T2DM), as T2DM is one of the most common chronic diseases among the elderly in China, and it is one of the two chronic diseases for disease management pilot in the Equalization of Essential Public Health Service program. The patients are generally old and not good at using smartphone. To ensure the quality, we could not collect data from patients by online survey. Considering feasibility, we could not recruit patients in all 60 CHSs in the intervention and control groups.

 Twelve CHSs were selected from the 30 CHSs in the intervention group and another 12 CHSs were selected from the 30 CHSs in the control group by simple random sampling (random number table method). Twenty patients with T2DM in each of the 24 CHSs were invited to participate in the survey through systematic sampling. Finally, 1901 care professionals and 532 patients with T2DM consented to participate in the study.

###  Data Collection

 The primary outcome was integrated care, measured by the Chinese version Rainbow Model of Integrated Care Measurement Tool (C-RMIC-MT). The RMIC-MT has been widely used to evaluate integrated care in Singapore, Australia, and the Netherlands,^19-21^ and the C-RMIC-MT was used to measure integrated care from the perspectives of both care professionals and patients. In the care professional version of the C-RMIC-MT (C-RMIC-MT-S), integrated care were evaluated in 6 dimensions: person-centerd & community-based, care integration, professional integration, organizational integration, technical competence, and cultural competence.^23^ In the patient version of the C-RMIC-MT (C-RMIC-MT-P), integrated care were evaluated in 5 dimensions: person-centeredness, clinical integration, professional integration, team-based coordination, and organizational integration.^22^ All items were answered on a 5-point Likert scale ranging from 1 to 5 (1 being strongly disagree, 5 being strongly agree). Total score of the whole scale and scores of each dimension were calculated by the sum score of included item. Additionally, we collected data about patients’ socio-demographic characteristics (gender, age, marital status, education level, employment status, income, and health status) and utilization of T2DM services (years with diabetes, diabetes complication, contract with family physician teams, and frequency of visits), care professionals’ socio-demographic characteristics (gender, age, and education level) and occupational factors (years of working, title, profession, patient volume, and team relationships).

 Baseline surveys were collected in June of 2018, and follow-up surveys were collected in July of 2021. Care professionals completed the online questionnaire, and patients with T2DM completed the paper questionnaire face to face with trained investigators. The study complied with the recommendations of the Declaration of Helsinki and was approved by the Ethical Committee of Public Health, SUN Yat-Sen University.

###  Data Analysis

####  Propensity Score Matching

 Propensity score matching (PSM) method proposed by Rosenbaum and Rubin was used to reduce the bias caused by confounding factors between the control group and the intervention group.^24^ The PSM model is given by the following:


(1) $$P_{i}\left(X\right)=\mathrm{Pr}\left({\mathrm{int}}_{it}=1\left|X_{i}\right.\right)=F\left[f\left(X_{i}\right)\right]$$

In equation (1), int_it_ = 1 represents the intervention group, and X_i_ represents the set of covariates. (X_i_) is a linear function, and F[f(X_i_)] is a logit function. The logit model was used to calculate the probability of sample *i* being included in the hospital group through a set of covariates, namely, the propensity score P_i_ (X). For care professionals, four factors (education level, professions, team relationship, and patient volume) with statistically significant between the two groups were included in the logit model to predict propensity scores. For patients, we preformed the estimation of the propensity scores by using the following variables with statistically significant between the two groups: gender, age, marital status, education level, and employment status. Then, we used one-to-one nearest neighbour matching, excluded observations not on the common support from the analysis. Finally, we assessed the reliability of the matching results by examining the balance of means of covariates between the intervention and control groups and the reduced standardized bias. After matching, there should be no statistically significant differences in the mean of covariates between the two groups (*P* > .05), and the standardized bias should be ≤5%.^25^

####  Difference-in-Differences Model

 After PSM matching, the control group and the intervention group had similar characteristics before the intervention, which satisfied the parallel trend hypothesis.^26^ The following equation of the difference-in-differences (DID) model is estimated for continuous outcomes:


(2) $$Y_{it}=\alpha +\beta_{1}{\mathrm{int}}_{i}+\beta_{2}\;year_{t}+\beta_{3}\left({\mathrm{int}}_{i}\times year_{t}\right)+\gamma X_{it}+\varepsilon_{it}$$

In equation (2), Y_it_ refer to the scores and total scores of various dimensions of integrated care evaluated by care professionals (patients with T2DM) at time *t*. Int_i_ is a grouping dummy variable, with int_i_ = 0 represents the control group, and int_i_ = 1 represents the intervention group. Year_t _represents the time dummy variable, with year_t _= 0 means the time before the establishment of the hospital group (June 2018), and year_t _= 1 means the time after the establishment of the hospital group (July 2021). The variable int_i_× year_t_ denotes the interaction between groups and time. *β*_1_ refers to the difference between control and intervention group before establishment of the hospital group, *β*_2_ denotes change in the score of integrated care in the control group after establishment of the hospital group, *β*_1_+*β*_3_ shows the difference between control and intervention group after the establishment of the hospital group. *β*_3_ represents the difference of difference in integrated care between control and intervention group (net intervention effect). *X*_it_ represents a set of individual covariates of care professionals (patients with T2DM) *i* at time *t*, ie, patients’ socio-demographic characteristics and utilization of T2DM services, and care professional’ demographic characteristics, socioeconomic characteristics and occupational factors, and *ε*_it_ is the error term. We estimated equation(2) using the ordinary least squares method. All statistical analyses were performed using Stata (version 16).

## Results

###  Results of Care Professionals’ Evaluation

 Table S1 presents the characteristics of the 1901 care professionals who participated in this study. The average scores of integrated care (all 6 dimensions) evaluated by care professionals in the intervention and control groups were 4.06 and 4.03 in 2018. And the scores increased to 4.18 and 4.08 in 2021, respectively. Figure (panel a) shows the scores for each dimension of the C-RMIC-MT-S.

**Figure:**
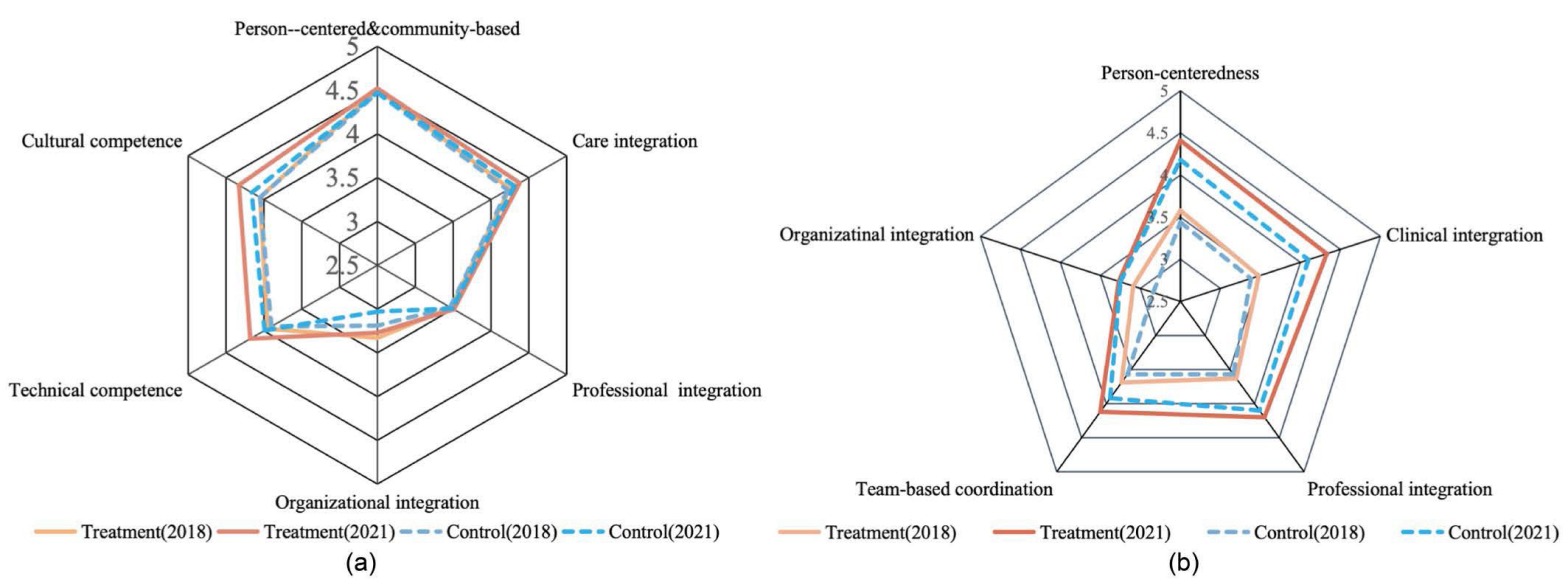
Average Scores for Each Dimension of the C-RMIC-MT-S and C-RMIC-MT-P. Abbreviations: C-RMIC-MT-S, care professional version of the C-RMIC-MT (Chinese version Rainbow Model of Integrated Care Measurement Tool); C-RMIC-MT-P, patient version of the C-RMIC-MT.

####  Balancing test for Propensity Score Matching 

 After PSM, 5 care professionals were excluded from the analysis. Table 1 shows that the differences of educational level, professions, team relationship, and patient volume between the intervention group and the control group were not statistically significant after matching. Moreover, the biases of the above four covariates were reduced to less than 5%, indicating that distributions of all four covariates between the two groups were consistent.

**Table 1:** Propensity Score Matching Data Balancing Test (Care Professionals and Patients With Type 2 Diabetes Mellitus)

**Variable**	**U/M**	**Mean**		**%Bias**	**%Reduct |Bias|**	***T***** Test**	
		**Treated**	**Control**			***t***	***P*** **> |*****t*****|**
Care professionals							
Education level	U	2.735	2.821	-14.3		-3.12	.002
	M	2.737	2.751	-2.3	84.2	-0.75	.453
Professions	U	1.879	2.014	-17.2		-3.74	<.001
	M	3.537	3.456	-0.0	100.0	0.00	1.000
Team relationship	U	1.050	1.092	-16.3		-3.52	<.001
	M	1.050	1.043	2.6	84.0	0.67	.503
Patient volume	U	2.600	2.455	10.6		2.31	.021
	M	2.599	2.586	0.9	91.4	0.19	.849
Patients							
Gender	U	1.369	1.456	-17.7		-2.04	.042
	M	1.371	1.363	1.5	91.4	0.18	.858
Age	U	2.019	2.448	-46.0		-5.31	<.001
	M	2.015	2.019	0.4	99.1	-0.05	.962
Marital status	U	1.055	1.130	-26.0		-3.01	.003
	M	1.049	1.049	0.0	100.0	-0.00	1.000
Education level	U	1.469	1.717	-28.9		-3.34	.001
	M	1.461	1.464	0.4	98.5	-0.06	.952
Employment status	U	2.052	1.858	26.3		3.04	.002
	M	2.052	2.052	0.0	100.0	-0.00	1.000

Abbreviations: U, Unmatched; M, Matched.

####  Propensity Score Matching and Difference-in-Differences Regression Estimation

Table 2 presents the outcome of PSM and DID regression. When covariates were not in the model, the establishment of the hospital group led to a positive increase in the assessment of all dimensions of integrated care by care professionals, and these effects were statistically significant on two dimensions. Compared with the control group, care professionals in the hospital group increased their evaluation of technical competence by 0.732 and their evaluation of cultural competence by 1.393. After adopting care professionals’ socio-demographic characteristics and occupational factors in the models, the results do not change. The assessment of technical competence by providers in the hospital group increased by 0.771, and the assessment of cultural competence increased by 1.423, compared to the control group.

**Table 2:** Propensity Score Matching and Difference-in-Differences Regression Estimates (Care Professional)

**Independent Variables**	**Dependent Variables**						
	**Person-Community** **-Centeredness**	**Care** **Integration**	**Professional Integration**	**Organizational Integration**	**Technical Competence**	**Cultural Competence**	**Total** **Score**
**PSM-DID Estimates (Without Covariables in the Model) **							
Int_it×_year_it_	0.096 (0.563)	0.724 (0.894)	0.033 (0.490)	0.407 (0.404)	0.732^*^ (0.386)	1.393^**^ (0.578)	3.386 (2.297)
Int_it_	0.353 (0.445)	0.319 (0.707)	0.060 (0.388)	0.557^*^ (0.319)	0.223 (0.306)	-0.005 (0.457)	1.507 (1.816)
Year_it_	0.040 (0.382)	1.176^*^ (0.607)	1.157 (0.333)	-0.640^**^ (0.274)	0.401 (0.262)	0.869^**^ (0.392)	2.003 (1.558)
N	1896	1896	1896	1896	1896	1896	1896
**PSM-DID Estimates (Adjusted For Covariables) **							
Int_it×_year_it_	0.041 (0.562)	0.591 (0.888)	0.036 (0.482)	0.474 (0.403)	0.771^**^ (0.380)	1.423^**^ (0.561)	3.336 (2.247)
Int_it_	0.229 (0.444)	0.127 (0.703)	0.038 (0.381)	0.557^*^ (0.319)	0.146 (0.301)	-0.219 (0.444)	0.878 (1.779)
Year_it_	-0.430 (0.404)	0.467 (0.639)	-0.142 (0.347)	-0.579^**^ (0.290)	0.212 (0.273)	0.449 (0.404)	-0.023 (1.616)
N	1896	1896	1896	1896	1896	1896	1896

Abbreviation: PSM-DID, propensity score matching and difference-in-differences regression. Robust standard errors in parentheses;^*^P <.100; ^**^P <.050; ^***^P <.010. Total score represents the sum of all the scores of all items in the C-RMIC-MT-S.

####  Heterogeneity Analysis

 With the adjusted model, we further analyzed the effects of the hospital group on integrated care as evaluated by different provider groups. As shown in Table 3, compared with GPs and other professions, nurses’ assessment of integrated care increased by an average of 6.736. Meanwhile, care professionals with senior title assessed integrated care with an increase of 13.384.

**Table 3:** Heterogeneity Analysis Adjusting for Covariables (Care Professional)

**Variables**	**Sex**		**Professions**			**Title**		
	**Male**	**Female**	**GP**	**Nurse**	**Others**	** Primary**	**Junior**	** Senior**
int_it×_year_it_	-2.948 (4.533)	5.758^**^ (2.581)	1.534 (3.667)	6.736^**^ (3.413)	-0.766 (4.933)	3.610 (3.526)	1.842 (3.211)	13.384^*^ (7.345)
Int_it_	4.858 (3.567)	0.924 (2.048)	2.334 (2.879)	-1.188 (2.615)	-2.867 (5.441)	0.625 (2.763)	-1.984 (2.551)	-6.170 (5.892)
Year_it_	3.255 (3.295)	-1.219 (1.848)	2.291 (2.761)	-3.332 (2.446)	-2.867 (5.441)	-2.240 (2.501)	1.666 (3.211)	-2.867 (5.441)
N	534	1362	638	716	542	840	891	165

Abbreviation: GP, general practitioner. Robust standard errors in parentheses;^*^P <.100; ^**^P <.050; ^***^P <.010.

####  Robustness Test

 We replaced one-to-one nearest neighbor matching with calliper nearest neighbor matching to examine the robustness of the PSM-DID results. Table S2 shows that the int_it _× year_it_ interaction terms of technical competence and cultural competence remain statistically significant despite minor changes with coefficient values and standard errors.

###  Results of Patients’ Evaluation

 Table S3 presents the characteristics of the 532 patients who participated in the study.

 The average scores of integrated care (all 5 dimensions) evaluated by patients in the intervention and the control groups were 3.51 and 3.39 in 2018. And the scores increased to 4.14 and 3.96 in 2021, respectively. Figure (panel b) shows the average scores for each dimension of the C-RMIC-MT-P.

####  Balancing Test for Propensity Score Matching 

 After PSM, 4 patients were excluded from the analysis. We examined the balance property of each covariate between and the reduced sampling bias before and after matching. As shown in Table 1, the differences in gender, age, marital status, education level, and employment status between the intervention group and the control group were no longer statistically significant after matching. Moreover, the biases of the above five covariates were reduced to less than 5%, indicating distributions of all five covariates between the two groups were consistent.

####  Propensity Score Matching and Difference-in-Differences Regression Estimation

Table 4 shows the outcome of PSM-DID regressions. When covariates were not in the model, the hospital group did not effectively improve patients’ assessment of different dimensions of integrated care, and reduced patients’ assessment of organizational integration by 0.495. After adopting patients’ socio-demographic characteristics and utilization of T2DM services in the model, the results did not change. Compared with the control group, the score of organizational integration perceived by patients in hospital group decreased by 0.649. However, although not statistically significant, the score of clinical integration assessed by patients in the hospital group increased by 0.015.

**Table 4:** Propensity Score Matching Data Balancing Test Estimates (Patients With Type 2 Diabetes Mellitus)

**Independent Variables**	**Dependent Variables**					
	**Person-** **Centeredness**	**Clinical Integration**	**Professional Integration**	**Team-Based Coordination**	**Organizational Integration**	**Total ** **Score**
**PSM-DID estimates (Without Covariables in the Model) **						
int_it×_year_it_	0.167 (0.221)	0.488 (0.496)	0.002 (0.309)	0.114 (0.267)	-0.495^**^ (0.202)	0.277 (1.221)
int_it_	0.297^*^(0.163)	0.529 (0.366)	0.210 (0.228)	0.388^**^ (0.198)	0.478^***^ (0.144)	1.901^**^ (0.901)
year_it_	1.472^***^(0.157)	3.572^***^ (0.353)	1.603^***^ (0.220)	1.056^***^ (0.191)	0.823^***^ (0.147)	8.526^***^ (0.870)
N	528	528	528	528	528	528
**PSM-DID Estimates (Adjusted for Covariables) **						
int_it×_year_it_	-0.062 (0.236)	0.015 (0.536)	-0.169 (0.333)	-0.115 (0.287)	-0.649^***^ (0.217)	-0.981 (1.308)
int_it_	0.280^*^ (0.167)	0.435 (0.378)	0.118 (0.235)	0.288^**^ (0.202)	0.488^***^ (0.153)	1.609^*^ (0.922)
year_it_	1.473^***^ (0.169)	3.656^***^ (0.378)	1.593^***^ (0.238)	1.131^***^ (0.205)	0.846^***^ (0.155)	8.699^***^ (0.935)
N	528	528	528	528	528	528

Abbreviation: PSM-DID, propensity score matching and difference-in-differences regression. Robust standard errors in parentheses;^*^P <.100; ^**^P <.050; ^***^P <.010. Total score represents the sum of all the scores of all items in the C-RMIC-MT-P.

####  Heterogeneity Analysis

 With the adjusted model, we further analyzed the assessment of integrated care by patients based on various socio-demographic variables. Table 5 displays that the score of integrated care by middle-income patients (¥50 001-¥100 000) decreased by 9.217, whereas the score assessed by high-income patients (≥¥100 000) increased by 8.043. Furthermore, the score of integrated care rated by patients with diabetes (less than 1 year) decreased by 12.917.

**Table 5:** Heterogeneity Analysis Adjusting for Covariables (Patients With Type 2 Diabetes Mellitus)

**Variables**	**Age**				**Income (Per Year)**		
	**≤50**	**51-60**	**61-70**	**>70**	**≤¥50000**	**¥50001-¥100000**	**≥¥100000**
int_it×_year_it_	-3.388 (2.800)	5.005^**^ (2.444)	-3.439 (2.659)	-4.795 (3.534)	1.049 (1.441)	-9.217^***^ (3.316)	8.043^*^ (4.607)
Int_it_	2.440 (1.799)	-1.324 (1.777)	1.616 (1.885)	3.453 (3.018)	0.953 (1.059)	5.429^**^(2.576)	0.556 (3.345)
Year_it_	9.171^***^ (2.146)	6.096^***^ (1.895)	9.863^***^ (1.611)	10.122^***^(2.798)	7.967^***^ (1.086)	13.548^***^ (2.431)	7.012^*^ (3.584)
N	139	183	125	54	360	116	52
**Variables**	**Years With Diabetes**						
	**<1 years**		**1-5 years**		**6-10 years**	**>10 years**	
int_it×_year_it_	-12.917^**^ (4.955)		2.916 (1.903)		1.945 (3.054)	-4.467 (3.174)	
Int_it_	7.826^*^ (4.526)		0.998 (1.298)		0.567 (1.841)	1.827 (2.425)	
Year_it_	17.407^***^ (3.905)		6.859^***^ (1.465)		8.150^***^ (2.012)	9.536^***^ (1.973)	
N	59		244		111	114	

Robust standard errors in parentheses;^*^P <.100; ^**^P <.050; ^***^P <.010.

####  Robustness Test

 To ensure the robustness of the PSM-DID results, we replaced one-to-one nearest neighbor matching with calliper nearest neighbor matching. As presented in Table S4 (See Supplementary file 1), the results did not change, consistent with those estimations shown in Table 4.

## Discussion

###  Main Findings 

 To our knowledge, this is the first study to evaluate the effect of a hospital group on integrated care using a standardized measurement tool for integrated care in China. This study found that the routine strategies used to build integrated healthcare systems in Shenzhen led to improvements in patient and provider perceptions of integrated care. Moreover, the hospital group, as an organizational integration strategy, had a limited additional effect on integrated care over the above routine strategies.

 Evaluation by care professionals indicated the hospital group significantly improved the technical competence and cultural competence, which are important basis for achieving integrated care.^27,28^ The establishment of the hospital group has promoted partnerships across different-levels medical institutions, which resulted in more effective training activities, technical communication and newly developed skills.^29^ Therefore, the positive effect in the technical competence of the health systems has been perceived by care professionals. Meanwhile, the integrated management of administration and personnel in the hospital group fostered share norms, values and visions among care professionals.^28^ Moreover, compared to GPs, nurses perceived higher-level integrated care in the hospital group. This difference between the experience of nurses and GPs might indicate differences in responsibilities, workload, or incentive structures. Unlike other countries, GPs in China are the leaders of the family physician teams, and need to confront more coordination issues than other professions working in the teams.^30,31^

 Evaluation by patients indicated that, contrary to our hypothesis, the hospital group did not significantly improve integrated care compared with the control group. This demonstrated that strategies taken in the hospital group did not bring coordinated care to patients.^32^ Several studies implied potential reasons for small or no differences in patients’ perception of integrated care.^33-35^ Firstly, the inter-institutional coordination of health resources in the hospital group were not implemented well. Secondly, routine care in CHSs were also improved towards integrated care with multidisciplinary family physician teams. However, although not statistically significant, clinical integration evaluated by patients has improved, which may be due to the referral system in the hospital group. Moreover, economic status of patients influenced changes in their evaluation of integrated care. Middle-income patients’ evaluation of integrated care turned worse significantly, while high-income patients’ evaluation turned better significantly. High-income patient group may have higher socioeconomic status and health literacy, and are more concerned about their own health, thus they are more aware of interventions for integration implemented in the hospital group.^36^ Evaluation of patients who had diabetes (less than 1 year) on integrated care went worse that evaluation of the others. The rapid increasing demand of integrated care in the first year of diabetes and the slow response of health system might contribute to a low rating for a short period.

###  Implications for Policy and Practice 

 The principle findings of this study provide evidence for promoting integrated care in the hospital group by understanding how patients and care professionals evaluate integrated care. These findings might be helpful for other medical consortia in Chinese primary care systems as hospital group are rolled out.

 First, we suggest that as organizational integration is implemented, more attention needs to be devoted to professional, clinical and other integration strategies in the establishment of medical consortia in China. Different from international experience, most pilot initiatives in China begun to promote integrated care with organizational integration according to the backgrounds of the health systems. Organizational integration can address the structural and governance barriers encountered by integration. However, based on pilot initiatives in Europe, some researchers have suggested that effective care integration could be achieved without organizational integration and that more attention should be given to clinical integration and professional integration, which have direct effects on care professionals’ behavior.^37,38^ Second, shared decision-making and self-management support should be better embedded in integrated care of the hospital group. Care professionals should empower patients to participate in care delivery actively, in order to improve their awareness and evaluation of integrated care. Third, our results indicated that, as integrated care planning is implemented, it is necessary to provide additional support for GPs regarding their practice and well-being. Janse also found that integrated care inevitably brings additional burdens to GPs.^39^ Finally, the achievement of integrated care is considered to be a long-term process, with continuous evaluation and policy adjustment. Rutten-van Mölken suggested that some EU-funded projects may need a minimum of five years to provide meaningful results.^40^

###  Strengths and Limitations 

 By using a standardized assessment tool, this study provides comparability with results from other studies locally and globally. Although this study was focused on the patients with T2DM, there are general implications for the field of integrated care. First, it provides the possibility for comparing results with studies using RMIC-MT in other countries. Although the C-RMIC-MT measures integrated care in different dimensions between patients and care professionals, its comprehensive evaluation could inform policy makers about how to strengthen people-centred integration and meet the needs of both patients and care professionals. Third, the PSM-DID regression approach addressed both the sample selectivity bias and individual heterogeneity and effectively estimated the net effect of the hospital group.^41^

 This study has some limitations. The first limitation is beyond the control of the research group. Driven by national policies of building people-centred integrated healthcare systems, Shenzhen has implemented a series of routine strategies for integrated care across the city since 2017. Therefore, all patient groups in Shenzhen have some exposure to integrated care. In future studies, we recommend using comparison groups from other cities or via longitudinal analysis for more accurate and robust measurements of treatment effects. Second, there is an institutional-individual hierarchy in patient data set. However, we did not collect institution-level information in the patient questionnaires. The fixed effects of institutions may have an impact on the evaluation of integrated care in hospital group. Third, COVID-19 might have affected respondents’ evaluation of integrated care. Strict lockdown and public health measures in China lowered patients’ access to routine diabetes care and follow-ups. Meanwhile, the provision of integrated care became difficult, as many medical resources of CHSs were purposed for epidemic prevention and control of the COVID-19.

## Conclusion

 The hospital group strategy was found to have limited additional effects on improvements in integrated care over and above the routine strategies taken in the control group. Therefore, it is necessary to pay attention to implementing comprehensive integration strategies to promote care integration in addition to organization integration strategies. Furthermore, the impacts of the hospital group on perception of patients and care professionals on integrated care depends on depend on population and healthcare-related characteristics such as education and provider profession. Further investigation regarding GPs’ experience with coordination in interprofessional teams and patients’ demands for people-centred care could potentially improve integrated care in hospital groups. Continuous longitudinal evaluation will be needed in the future to assess the long-term impacts of integrated strategies, to explore the pathways and mechanisms of these strategies and to provide evidence for refining policy for integrated care.

## Acknowledgements

 The authors thank all participants who provided valuable insights for the study.

## Ethical issues

 The protocol for the research project has been approved by Ethics Committee of School of Public Health, SUN Yat-Sen University (ref 2017 No.073).

## Competing interests

 Authors declare that they have no competing interests.

## Funding

 This work was supported by the National Natural Science Foundation of China (grant number 71804202).

## Supplementary files

Supplementary file 1 contains Tables S1-S4.
