# Supplementary material for: Patients’ and Care Professionals’ Evaluation of the Effect of a Hospital Group on Integrated Care in Chinese Urban Health Systems: A Propensity Score Matching and Difference-in-differences Regression Approach
Source: Int J Health Policy Manag. 2023 Nov 28;12:7897. doi: 10.34172/ijhpm.2023.7897 (PMC10843371; doi:10.34172/ijhpm.2023.7897)
Supplement: Supplementary file 1 — contains Tables S1-S4. [file ijhpm-12-7897-s001.pdf]

Article title: Patients' and Care Professionals' Evaluation of the Effect of a Hospital Group on Integrated Care in Chinese Urban Health Systems: A Propensity Score Matching and Difference-inDifferences Regression Approach

Journal name: International Journal of Health Policy and Management (IJHPM)

Authors' information: Xin Wang[1], Caiyun Zheng[1], Yao Wang[1], Stephen Birch[2], Yixiang Huang[1]*, Pim Valentijn[3,4]

[1]School of Public Health, Sun Yat-Sen University, Guangzhou, China.

[2]Centre for the Business and Economics of Health, University of Queensland, Brisbane, QLD, Australia.

[3]Department of Health Services Research, Care and Public Health Research Institute (CAPHRI), Faculty of Health, Medicine and Life Sciences, Maastricht University, Maastricht, The Netherlands.

[4]Essenburgh Research & Consultancy, Essenburgh Group, Harderwijk, The Netherlands.

***Correspondence to:** Yixiang Huang; Email: huangyx@mail.sysu.edu.cn

Citation: Wang X, Zheng C, Wang Y, Birch S, Huang Y, Valentijn P. Patients' and care professionals' evaluation of the effect of a hospital group on integrated care in Chinese urban health systems: a propensity score matching and difference-in-differences regression approach. Int J Health Policy Manag. 2023;12:7897. doi:10.34172/ijhpm.2023.7897

Supplementary file 1

Tables S1-S4.

Table S1 Characteristics of care providers

Demographic characteristic	2018		2021	
	Treatment N=323 *n*(%)	Control N=392 *n*(%)	Treatment N=560 *n*(%)	Control N=626 *n*(%)
Gender				
Male	99(30.65)	106(27.04)	161(28.75)	171(27.32%)
Famale	224(69.35)	286(72.96)	399(71.25)	455(72.68%)
Age				
30 years and under	59(18.27)	80(20.41)	177(31.61)	201(32.11%)
31-40 years old	147(45.51)	201(51.28)	219(39.11)	248(39.62%)
41-50 years old	100(30.86)	98(25.00)	131(23.39)	152(24.28%)
over 50 of age	17(5.26)	13(3.32)	33(5.89)	25(3.99%)
Education level				
Senior high school and below	17(5.26)	10(2.55)	18(3.21)	11(1.76%)
College	88(27.24)	107(27.30)	120(21.43)	115(18.37%)
Undergraduate	213(65.94)	258(65.82)	383(68.39)	435(69.49%)
Graduate	5(1.55)	17(4.34)	39(6.96)	65(10.38%)
Years of working				
<5	42(13.00)	65(16.58)	201(35.89)	190(30.35)
5-10	61(18.89)	76(19.39)	95(16.96)	137(21.88)
>10	220(68.11)	251(64.03)	264(47.14)	299(47.76)
Title				
Primary	138(42.72)	190(48.47)	234(41.79)	280(44.73)
Junior	152(47.06)	171(43.62)	275(49.11)	295(47.12)
Senior	33(10.22)	31(7.91)	51(9.11)	51(8.15)
Professional				
General practitioner	118(36.53)	127(32.40)	211(37.68)	183(29.23)
Nurse	131(40.56)	167(42.60)	201(35.89)	217(34.66)
Others[#]	74(22.91)	98(25.00)	148(26.43)	226(36.10)
Patient volume				
<1000	242(74.92)	297(75.77)	212(37.86)	271(43.29)
1000-1999	55(17.03)	55(14.03)	273(48.75)	291(46.49)
≧2000	26(8.05)	40(10.20)	75(13.39)	64(10.22)
Team relationships				
Harmony	302(93.50)	346(88.27)	538(96.07)	579(92.49)
General/Not sure	21(6.50)	46(11.73)	21(3.75)	46(7.35)
Not harmonious	0(0.00)	0(0.00)	1(0.18)	1(0.16)

[#]Others include specialist, public health physician, pharmacist, laboratory worker, traditional Chinese medicine physician and health manager.

Table S2 Robustness test of PSM-DID estimation with caliper nearest neighbor matching (care professional)

Independent variables	Dependent variables						
	Person-community -centeredness	Care integration	Professional integration	Organizational integration	Technical competence	Cultural competence	Total score
$int_{it} \times year_{it}$	0.057	0.586	0 .004	0.481	0.760**	1.426**	3.315
	(0.562)	(0.887)	(0.482)	(0.404)	(0.381)	(0.562)	(2.245)
int_{it}	0.205	0.105	0.047	0.555*	0.218	-0.225	0.831
	(0.444)	(0.702)	(0.382)	(0.319)	(0.274)	(0.445)	(1.777)
$year_{it}$	-0.458	0.451	-0.114	-0.586**	0.218	0.443**	-0.046
	(0.404)	(0.638)	(0.347)	(0.291)	(0.301)	(0.405)	(1.617)
N	1891	1891	1891	1891	1891	1891	1891

Robust SEs in parentheses; $^{*}p < 0.10$; $^{**}p < 0.05$; $^{***}p < 0.01$.

Total score represents the sum of all the scorse of all items in the C-RMIC-MT-S.

Table S3 Characteristics of patients

Demographic characteristic	2018 Treatment N=128 *n*(%)	2018 Control N=117 *n*(%)	2021 Treatment N=143 *n*(%)	2021 Control N=144 *n*(%)
Gender				
Male	90(70.31)	60(51.28)	81(56.64)	82(56.94)
Famale	38(29.69)	57(48.72)	62(43.36)	62(43.06)
Age				
50 years and under	51(39.84)	25(21.37)	39(27.27)	26(18.06)
51-60 years old	43(33.59)	29(24.79)	64(44.76)	47(32.64)
61-70 years old	25(19.53)	45(38.46)	28(19.58)	55(38.19)
over 70 of age	9(7.03)	18(15.38)	12(8.39)	16(11.11)
Marital status				
Married	122(95.31)	104(88.89)	134(93.71)	123(85.42)
Others[#]	6(4.69)	13(11.11)	9(6.29)	21(14.58)
Education level				
Junior high school and below	80(62.50)	70(59.83)	97(67.83)	76(52.74)
Senior high school	34(26.56)	28(23.93)	31(21,68)	40(27.78)
College	10(7.81)	9(7.69)	15(10.49)	13(9.03)
Undergraduate	4(3.13)	10(8.55)	0(0.00)	15(10.42)
Employment status				
Retired from paid work	23(17.97)	41(35.04)	28(19.58)	65(45.14)
Employed	79(61.72)	34(29.06)	76(53.15)	52(36.11)
Others[†]	26(20.31)	42(35.90)	39(27.27)	27(18.75)
Income(per year)				
≤50,000	97(75.78)	91(77.78)	86(60.14)	90(62.50)
60,000-100,000	18(14.06)	19(16.24)	42(29.37)	37(25.69)
≥110,000	13(10.16)	7(5.98)	15(10.49)	17(11.81)
Health status				
Very good and good	98(76.56)	83(70.94)	133(93.01)	106(73.61)
Fair	24(18.75)	32(27.35)	13(9.09)	38(26.39)
Poor and very poor	6(4.69)	2(1.71)	3(2.10)	1(0.69)
Years with diabetes				
≤5 years	80(62.50)	49(41.88)	94(65.73)	81(56.25)
6-10years	32(25.00)	37(31.62)	18(12.59)	26(18.06)
>10 years	16(12.50)	31(26.50)	31(21.68)	37(25.69)
Diabetes complication				
Yes	11(8.59)	21(17.95)	19(13.29)	44(30.56)
No	117(91.41)	96(82.05)	124(86.71)	100(69.44)
Contract with family doctor teams				
Yes	34(26.56)	28(23.93)	122(85.31)	117(81.25)
No	94(73.44)	89(76.07)	21(14.69)	27(18.75)
Frequency of visits(per month)				
<1 visits	31(24.22)	43(36.75)	38(26.57)	48(33.33)
1 ~ 2 visits	93(72.66)	71(60.68)	95(66.43)	90(62.50)
≥ 3visits	4(3.13)	3(2.56)	10(6.99)	6(4.17)

[#]Others consist of Unmarried, divorced and widowed. [†]Others consist of unemployed for taking care of family, for illness and for no reasons.

Table S4 Robustness test of PSM-DID estimation with caliper nearest neighbor matching (patients)

Independent variables	Dependent variables					
	Person-centeredness	Clinical integration	Professional integration	Team-based coordination	Organizational integration	Total score
$int_{it} \times year_{it}$	-0.093	-0.079	-0.171	-0.072	-0.654***	-1.069
	(0.242)	(0.549)	(0.341)	(0.294)	(0.206)	(1.343)
int_{it}	0.291*	0.464	0.112	0.265*	0.489***	1.622*
	(0.168)	(0.382)	(0.237)	(0.205)	(0.155)	(0.933)
$year_{it}$	1.507***	3.743***	1.586***	1.085***	0.850***	8.771***
	(0.175)	(0.398)	(0.247)	(0.213)	(0.162)	(0.973)
R^2	0.3227	0.3446	0.2041	0.1764	0.1130	0.3225
N	517	517	517	517	517	517

Robust standard errors (SEs) in parentheses; $^{*}p < 0.10$; $^{**}p < 0.05$; $^{***}p < 0.01$.

Total score represents the sum of all the scorse of all items in the C-RMIC-MT-P.
